# Topoisomerase 3α and RMI1 Suppress Somatic Crossovers and Are Essential for Resolution of Meiotic Recombination Intermediates in *Arabidopsis thaliana*

**DOI:** 10.1371/journal.pgen.1000285

**Published:** 2008-12-19

**Authors:** Frank Hartung, Stefanie Suer, Alexander Knoll, Rebecca Wurz-Wildersinn, Holger Puchta

**Affiliations:** Botany II, University of Karlsruhe, Karlsruhe, Germany; National Cancer Institute, United States of America

## Abstract

Topoisomerases are enzymes with crucial functions in DNA metabolism. They are ubiquitously present in prokaryotes and eukaryotes and modify the steady-state level of DNA supercoiling. Biochemical analyses indicate that Topoisomerase 3α (TOP3α) functions together with a RecQ DNA helicase and a third partner, RMI1/BLAP75, in the resolution step of homologous recombination in a process called Holliday Junction dissolution in eukaryotes. Apart from that, little is known about the role of TOP3α in higher eukaryotes, as knockout mutants show early lethality or strong developmental defects. Using a hypomorphic insertion mutant of *Arabidopsis thaliana* (*top3α-2*), which is viable but completely sterile, we were able to define three different functions of the protein in mitosis and meiosis. The *top3α-2* line exhibits fragmented chromosomes during mitosis and sensitivity to camptothecin, suggesting an important role in chromosome segregation partly overlapping with that of type IB topoisomerases. Furthermore, AtTOP3α, together with AtRECQ4A and AtRMI1, is involved in the suppression of crossover recombination in somatic cells as well as DNA repair in both mammals and *A. thaliana*. Surprisingly, AtTOP3α is also essential for meiosis. The phenotype of chromosome fragmentation, bridges, and telophase I arrest can be suppressed by At*SPO11* and At*RAD51* mutations, indicating that the protein is required for the resolution of recombination intermediates. As At*rmi1* mutants have a similar meiotic phenotype to At*top3α* mutants, both proteins seem to be involved in a mechanism safeguarding the entangling of homologous chromosomes during meiosis. The requirement of AtTOP3α and AtRMI1 in a late step of meiotic recombination strongly hints at the possibility that the dissolution of double Holliday Junctions via a hemicatenane intermediate is indeed an indispensable step of meiotic recombination.

## Author Summary

The topoisomerases of the class IA are present in all three eukaryotic kingdoms—plants, fungi, and animals—and are involved in DNA replication and DNA repair. During the course of their action, they introduce transient single-strand nicks into DNA. In higher eukaryotes, two different classes of the enzymes are present: TOP3α and TOP3β. TOP3α is essential, as disruption of its function usually results in lethality of the affected organism. Using a mutant of TOP3α that retains some activity, we show that the protein has multiple, different functions in the model plant *A. thaliana*. Besides its action in somatic cells, where it is required for mitosis as well as DNA repair, we demonstrate that TOP3α together with its protein partner RMI1 is essential for meiosis. Here, both proteins are involved in DNA recombination—the exchange of information between parental chromosomes. Disruption of either TOP3α or RMI1 leads to grave defects and an early termination of meiosis, resulting in the sterility of the mutant plants. Our detailed analysis indicates that both proteins are involved in a late step of meiotic recombination, in a mechanism that prevents entanglement of the parental chromosomes. Thus, meiotic recombination seems to be more complex than previously anticipated.

## Introduction

Topoisomerases are enzymes with crucial functions in DNA metabolism. They are ubiquitously present in prokaryotes and eukaryotes and modify the steady-state level of DNA supercoiling [1]. Topoisomerases are required for cellular processes, such as DNA replication, transcription, recombination and chromatin remodeling to release topological forces by cleaving the DNA backbone in a reversible manner. Topoisomerases are sorted into two basic classes that differ in their ability to create either single strand (class I) or double strand breaks (class II). They can be subdivided into two classes each, which have been defined either by their chemical properties (IA and IB) or by structural differences between the enzymes (IIA and IIB) [1],[2]. There are three topoisomerases in yeast: TOP1, 2 and 3. In contrast to TOP1 and 2, which are well characterized and involved in DNA replication (TOP1, type IB) or decatenation of linked chromosomes (TOP2, type IIA), the role of TOP3 (type IA) is poorly understood. Mutants of *TOP3* in *Saccharomyces cerevisiae* show hyper-recombination, chromosome instability and do not sporulate, whereas *top3* mutants in *Schizosaccharomyces pombe* are lethal [3],[4]. In higher eukaryotes, two homologues of TOP3 were found and annotated as TOP3α and ß, respectively [5]–[7]. A knockout of *TOP3α* leads to early embryogenic lethality in mice and *Drosophila melanogaster* and to pleiotropic effects, such as germ cell proliferation abnormalities in *Caenorhabditis elegans* [8]–[10]. In contrast, mice mutated for TOP3ß do not exhibit lethality or growth abnormalities, but do possess a shortened lifespan [11]. We recently demonstrated that a T-DNA insertion line of Arabidopsis TOP3α (*top3α-1*) also exhibits severe developmental defects resulting in lethality of the plantlets [12]. In animals and plants, TOP3α proteins show an intimate physical and genetic interaction with one or more RecQ helicases. A tripartite protein complex indeed exists and consists of i) a RecQ helicase; ii) a TOP3α homolog and iii) a recently characterized protein named BLAP75 (for Blooms associated protein 75kd, in mammals) or Rmi1 (for RecQ mediated instability 1, in yeast) that mediates the complex formation of all three proteins [13]–[16]. This complex has also been referred to as the RTR or BTB complex (for RECQ/TOP3α/RMI or Blooms/TOP3α/BLAP75, respectively), and is able to dissolve recombination intermediates such as double Holliday Junctions (dHJs) or disrupt D-loop structures that otherwise lead to cell cycle arrest or cell death in different organisms [17]–[19]. Although ample evidence has accumulated on the biochemical properties of RTR *in vitro* and some of its functions in somatic cells, the role of the complex in meiosis has remained obscure. Using the model plant Arabidopsis, we demonstrate that the respective RTR homologues (RECQ4A, TOP3α and RMI1) have similar functions in somatic plant cells but that TOP3α as well as RMI1 are also required for the resolution of meiotic recombination intermediates.

## Results

### Phenotypes of the T-DNA Insertion mutants *top3α-1* and *2*

**Figure 1:**
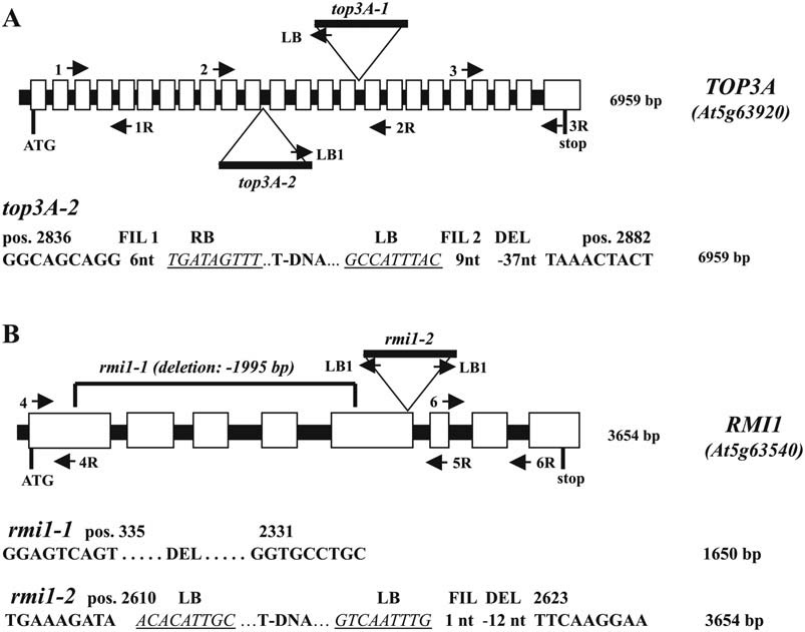
Molecular analysis of the T-DNA insertion lines. (A) The location of the T-DNA insertion in lines *top3α-1* and *2* is depicted. The schematically drawn *TOP3α* gene (At5g63920) contains 24 exons (white boxes). The T-DNA insertions interrupted the gene either in intron 16 (*top3α*-1) or in intron 11 (*top3α-2*) (B) Scheme of genomic changes of *rmi1*-1 and *2* mutants. At*RMI1* (At5g63540) contained eight exons, *rmi1-1* showed a 2 kb deletion including exon 1-5 and in *rmi1-2* the T-DNA is inserted into the 5th exon. Primers used are indicated by numbered or labelled arrows. The total length of the original or truncated gene (in case of *rmi1-1*) is given on the right. The genomic sequences adjacent to each T-DNA insertion or deletion locus were determined by PCR and sequencing, and are depicted below the schematic drawings. The genomic sequences are shown in bold and the respective left or right border sequences are in italics and underlined. Abbreviations: LB = left border; RB = right border; FIL = filler; DEL = deletion.

To elucidate the biological role of TOP3α we characterized two different T-DNA insertion lines named *top3α-1* (SALK_139357) and *top3α-2* (GABI_476A12) of the respective gene At5g63920 [20],[21]. The sequences of the insertion site of *top3α-1* have been previously described [12]. The T-DNA of *top3α-2* is inserted into the 11th intron (of a total of 23) accompanied by a genomic deletion of 37 bp and two small filler sequences (Figure 1A). The nucleotide sequence of the mRNA of At*TOP3α* was determined by RT-PCR and RACE [22] and deposited into GenBank (acc. no. EU295446). The T-DNA insertions of both lines are shown in Figure 1A and are located in the mid-region of the gene. No expression of the gene spanning the respective T-DNA insertion sites could be detected (Figure S1A; Table S1).

**Figure 2:**
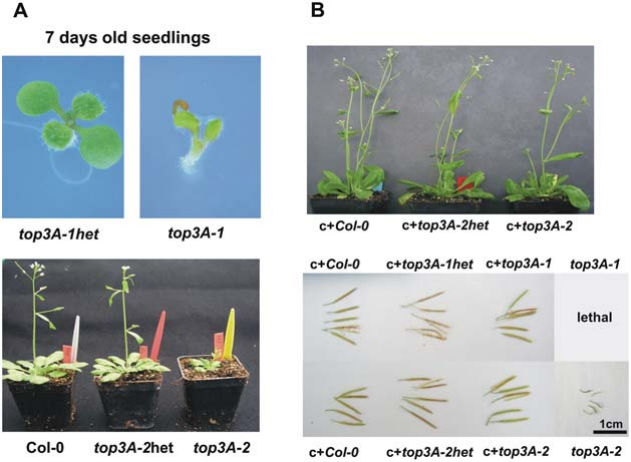
Phenotypes of the *top3α-1* and *2* mutant lines and the complemented plants. (A) Upper panel: Typical example of a homozygous *top3α-1* seedling on agar plates shown seven days after germination. A normally growing heterozygous seedling is shown on the left for comparison. Lower panel: Examples of wild type (left), heterozygous (middle) and homozygous (right) *top3α-2* plants after four weeks grown in soil. The heterozygous plant appeared absolutely normal, whereas the homozygous plant was dwarfed and showed malformed or fasciated organs. (B) Upper panel: Wild type Col-0, heterozygous and homozygous *top3α-2* mutant plants each containing the complementation construct (c+). Plants were transferred into soil after three weeks being on gentamycin selection plates. All three types of successfully transformed plants appeared normal and yielded comparable amounts of seeds. Lower panel: Exemplary siliques of the plants transformed with the complementation construct in different genetic backgrounds (wild type, heterozygous or homozygous for the respective T-DNA insertion) and from the non-transformed *top3α-2* line are shown.

At*top3α-1* shows severe developmental defects and barely germinates; the mutant has deformed cotyledons and is not able to form roots at all (Figure 2A, upper panel). As demonstrated previously, this early lethality can be converted to a less severe phenotype in an At*recq4A-4* background [12]. The phenotype of the second line, At*top3α-2,* is less severe but nevertheless, visibly exhibits growth deformations, such as dwarfing, curling and fasciated organs as well as sterility (Figure 2A, lower panel). In their respective heterozygous mutant states, neither TOP3α insertion lines are visibly affected, and they can be propagated for at least four generations in our hands. In analyzing more than 40 homozygous *top3α-2* plants, a single intact seed was never observed. Interestingly, this phenotype is very similar to the one obtained for the double mutant *recq4A-4/top3α-1.*

The weaker phenotype of the *top3α-2* mutant indicates that this mutation is hypomorphic and allows somatic growth. Nevertheless, mitosis is severely impaired in this mutant. While analyzing mitotic divisions by DAPI staining, we found mitotic aberrations in 14.2% of the dividing cells, which is more than ten times higher than in the wild type Col-0 control (Table 1). Interestingly, we were able to detect a significantly similar level of mitotic errors in the *top3α-1/recq4A-4* or *top3α-2/recq4A-4* double mutants (15.6% and 10.5%, respectively, Table 1). The *recq4A-4* mutant behaved like the wild type, indicating that the protein is not required for proper mitosis.

**Table 1:** Frequency of mitotic defects in different T-DNA insertion lines.

line	counted stages	defective stages	[%]
Col-0	328	4	1.2
*recq4A-4*	574	4	0.7
*top3α-2*	539	78	14.5
*top3α-2/recq4A-4*	200	21	10.5
*top3α-1/recq4A-4*	422	66	15.6
*rmi1-1*	435	5	1.1
*rmi1-2*	484	4	0.8

Mitotic stages of the different lines and the control Col-0 have been counted from 2 to 3 independent preparations. According to Fisher's exact test, the double mutants are significantly different from the wild type at a significance level of p<0.001 using a confidence interval of 99% and they are not significantly different from *top3α-2* alone.

### Complementation of the Phenotypes of Both *top3α* Mutants

Using the full-length genomic sequence, we were able to complement the strikingly different phenotypes of both TOP3α insertion lines. A genomic fragment containing the complete gene including the promoter and terminator region was amplified, cloned into a binary vector and transformed into both heterozygous mutant lines via *Agrobacterium tumefaciens* using the floral dip method [23]. We tested plants homozygous for the T-DNA insertion in the *TOP3α* gene containing the complementation construct. For *top3α-1*, two out of two and for *top3α-2*, five out of five tested independent transformants showed a virtually complete rescue of all phenotypic traits: complemented *top3α-1* as well as *top3α-2* plants grew properly without fasciated organs and were as fertile as the wild-type (Figure 2B). This analysis demonstrates that the phenotypes are indeed caused by the two different insertions in the TOP3α gene.

### Sensitivity to Genotoxic Agents

**Figure 3:**
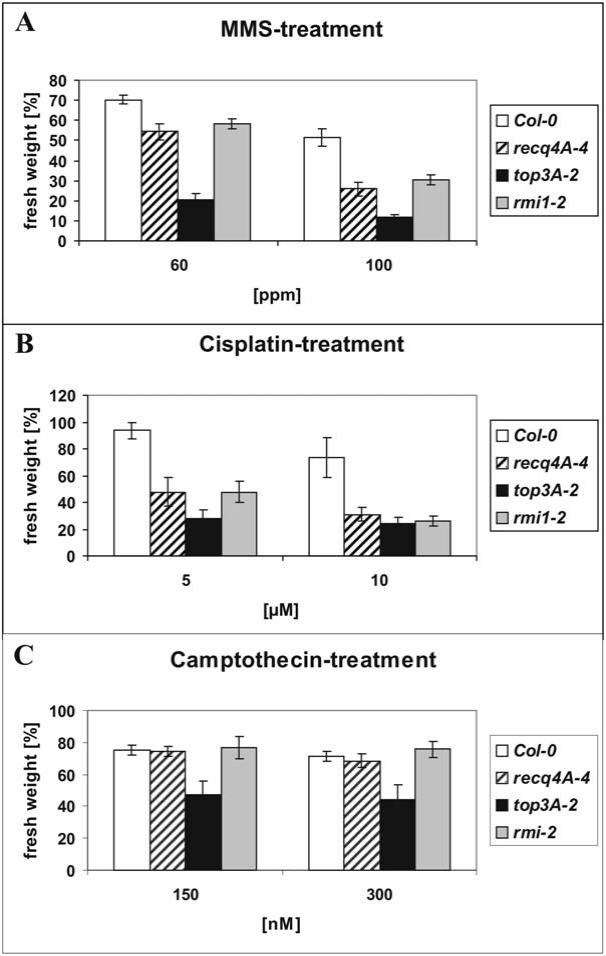
Mutagen sensitivity assays using MMS, cisplatin or camptothecin. (A to C) Fresh weight of five plantlets from each mutagen concentration was determined and expressed in percentage. The percentages were calculated from the relation of fresh weight of each line at a given mutagen concentration to the fresh weight of the same line without mutagen. Each assay was performed at least five times as described. Mean values and standard deviations are given. All three lines (*recq4A-4*, *top3α-2* and *rmi1-2*) were sensitive to MMS and cisplatin, whereas only *top3α-2* showed a significant sensitivity towards camptothecin (panel C). *Top3α-2* also showed a more elevated MMS sensitivity than *recq4A-4* or *rmi1-2* (panel A). ppm = parts per million.

To further characterize the role of TOP3α in Arabidopsis, we analyzed the DNA repair capacity of the *top3α-2* line. As TOP3α and BLM act together in the RTR complex in mammals, we included an insertion mutant of the RECQ4A gene in the analysis, which in many respects can be regarded as a functional BLM homologue in Arabidopsis [12],[24]. We applied a liquid medium assay and determined the weight of 4-week-old plantlets challenged with different concentrations of DNA damaging agents. None of the analyzed insertion lines showed an altered sensitivity towards bleomycin (data not shown). Compared to Col-0 wild types, *recq4A-4* and *top3α-2* were more impaired in growth when challenged with either the genotoxic agent methylmethane sulfonate (MMS) or the crosslinking agent cisplatin (Figure 3A and B). Furthermore, *top3α-2* was more sensitive to the topoisomerase I inhibitor camptothecin (CPT) while *recq4A-4* was not (Figure 3C).

### Homologous Recombination

Because the RTR complex plays an important role in the suppression of crossover (CO) recombination in somatic cells, we tested whether At*top3α-2* could be characterized by an enhanced frequency of somatic homologous recombination as was previously demonstrated for several At*RECQ4A* insertion mutants [12],[25]. For this purpose, we used the recombination substrate line IC9C. This line harbours a transgene with non-functional overlapping parts of the ß-glucuronidase gene. Restoration of the marker is possible only by using the sister chromatid or the homologous chromosome as a partner [26]. Each recombination event is represented as a blue stained sector. We determined the HR frequencies of the different mutant lines with and without the bleomycin challenge as a means of inducing double stranded breaks (DSBs).

**Figure 4:**
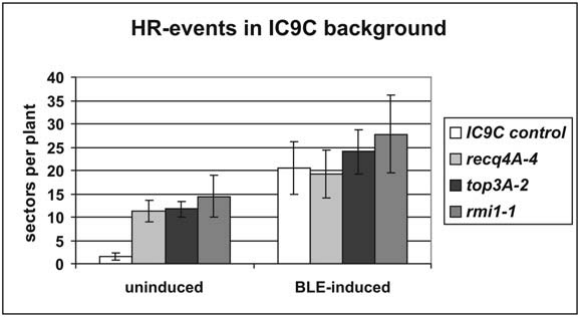
Recombination frequencies of untreated and bleomycin treated T-DNA insertion lines. Mean value of at least five independent assays using the IC9C background line in which restoration of the functional GUS-gene was only possible by intermolecular recombination. All three T-DNA insertion lines (*recq4A-4, top3α-2* and *rmi1-1*) showed a five to seven-fold enhanced basic level of crossovers. The absolute numbers of blue sectors after induction of DSBs by bleomycin were more or less the same in all three lines. This indicates that the RTR complex is not involved in intermolecular DSB repair but rather in replication-dependent recombination.

The HR frequency of both mutant lines (*top3α-2* and *recq4A-4*) in the IC9C background was enhanced to a similar extent (five- to seven-fold, respectively; Figure 4). After induction of DSBs, however, the enhancement could no longer be detected in either mutant lines compared to wild types. This finding reflects the behaviour of the *recq4A* mutant. One possible explanation would be that RECQ4A and TOP3α are involved in replication-associated crossover-suppression, but are not required for DSB-induced intermolecular HR. According to the SDSA model, which is appropriate for the description of homologues DSB repair in somatic plant cells, no crossovers should occur [27]. Alternatively, RECQ4A and TOP3α might prevent replication failures that would initiate recombination.

### Analysis of the Meiotic Defects in At*top3α-2*

**Figure 5:**
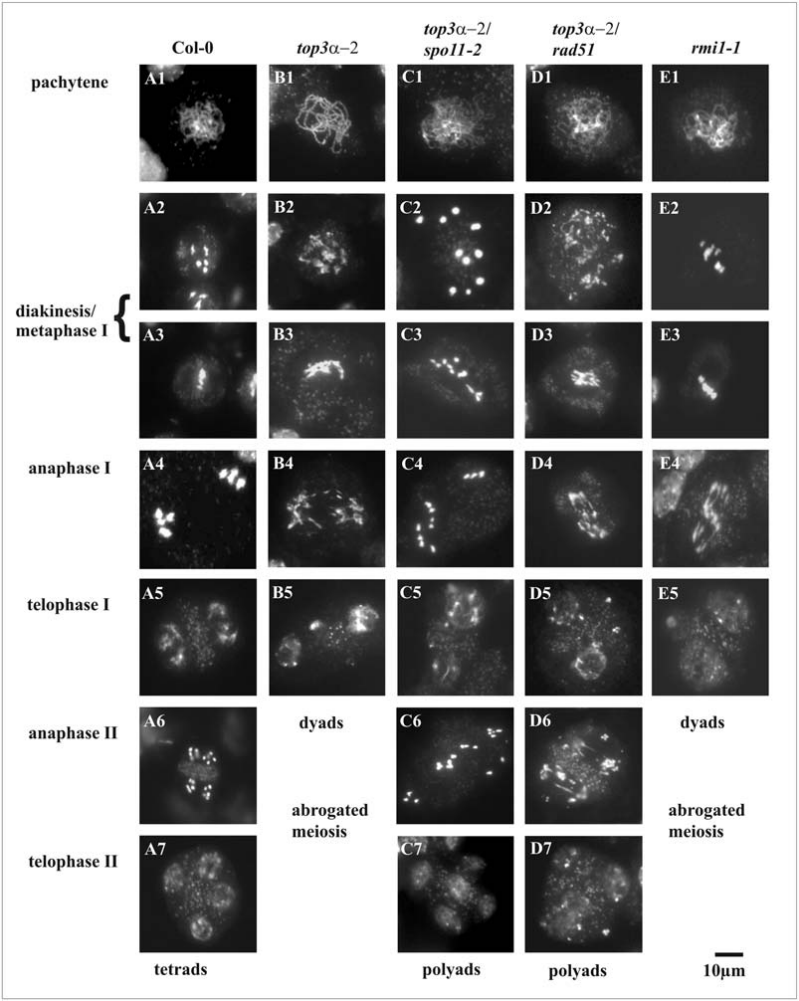
DAPI staining of different meiotic stages during pollen development in the mutant lines. Wild type meiosis (A1–7) is characterized by synapsis of homologous chromosomes and the formation of bivalents during the pachytene stage of prophase (A1). During diakinesis, the chromosome pairs condensed for the first meiotic division (A2). At metaphase I, homologues assembled at the metaphase plate (A3). During anaphase I, the homologous chromosomes separated to the poles (A4) and decondensed in telophase I (A5). During anaphase II, the chromatids slightly condensed and separated (A6), followed by another decondensation phase in telophase II and ended up in tetrads (A7). The mutant lines *top3α-2* and *rmi1-1* both exhibited chromosome fragmentation became visible in diakinesis and ending in telophase I (B2 to B5 and E2 to E5). Furthermore, both lines did not progress to meiosis II and showed an arrest in telophase I (B5 and E5). In the *rmi1-1* mutants, massive amounts of stainable DNA fragments appeared stuck at the metaphase plate (compare B4 to E4). Double mutants of *top3α-2* and two genes involved in early steps of meiotic DSB repair (At*spo11-2* and At*rad51*) showed the respective phenotype of the background mutant in both cases, indicating that *top3α-2* is involved in the latter steps of DSB repair (C1 to 7 and D1 to 7). The arrest in telophase I was released in *spo11-2* or *rad51* backgrounds, respectively.

The most striking phenotype of the *top3α-2* and the *recq4A-4/top3α-1* double mutant is their absolute sterility. To define the essential role of AtTOP3*α*, we analyzed the meiotic division of *top3α-2* in detail using DAPI staining of pollen mother cells (PMC). Representative meiotic stages of PMCs are shown in Figure 5. In the early stages of meiosis, the *top3α-2* mutant appears normal, and the first defects become visible during the late prophase when more than five bivalent structures appear, indicating the presence of breaks in the chromosomes (Figure 5 B2). In metaphase I, further chromosome condensation is disturbed and fragmentation becomes more visible (Figure 5 B3). The most impressive effect is observed in anaphase I: some chromosomal DNA is pulled to the poles but appears to stick together with other fragments that are, by themselves, not heading for the poles while some fragments stay in the former location of the metaphase plate (Figure 5 B4). After decondensation of the chromosomal fragments in telophase I, we were unable to observe the usual progress of meiosis towards the second division. This suggests that *top3α-2* is arrested at this stage (Figure 5 B5). We also analyzed *recq4A-4*, which did not show such defects and behaved like Col-0 during meiotic divisions in PMCs (Figure S2).

To further define the nature of DNA structures that are processed by TOP3α during meiosis, it was necessary to define whether the enzyme acts on replication or recombination intermediates, and in the latter case, what types of intermediates might be targeted. To test this, crosses of *top3α-2* with two lines defective in either initiation (At*spo11-2-3*) or further progression (At*rad51*) of meiotic recombination were analyzed [28],[29]. The At*spo11-2-3* mutant produces no visible meiotic DSBs and exhibits unpaired univalents which are randomly distributed during the meiotic division. If TOP3*α* is involved in the processing of recombination intermediates, we would expect that the *top3α-2* phenotype would be suppressed by the *spo11-2-3* mutation. This is indeed the case, as the combination *top3α-2/spo11-2-3* shows a clear *spo11-2-3* phenotype and no traces of chromosome fragmentation (Figure 5 C1 to C7). Furthermore, the arrest of *top3α-2* observed after the first meiotic division can be overcome in the *spo11-2* background (Figure 5 C6 and C7). The fact that TOP3*α* is required for a stage of meiotic recombination after strand invasion is revealed by the fact that the double mutant *top3α-2/rad51* has the same phenotype as the *rad51* mutant, which shows abnormal pachytene chromosomes (Figure 5 D1 to D7) [29]. Most notably in the double mutant, the second meiotic division takes place and results in polyads instead of the typical tetrads observed in the wild type (Figure 5 D6 and D7). Thus, TOP3*α* acts on DNA structures that are formed after DSB induction and heteroduplex invasion. The enzyme, therefore, appears to be involved in the resolution of a certain class of heteroduplex intermediates.

### Phenotype of the AtRMI1/BLAP75 Mutants *rmi1-1* and *2*

The RMI1/BLAP75 protein has been identified recently as the third partner of the RTR complex in mammals and yeast [17]. We therefore analyzed the Arabidopsis genome for a putative homologue of RMI1. We identified two candidate genes, At5g63540 (that we refer to below as AtRMI1) and At5g19950. As insertion mutants of At5g19950 did not reveal sensitivity to DNA damaging agents or reduced fertility (data not shown), we assumed that the putative ORF, in contrast to At5g63540, was not a functional homologue of RMI1. For At5g63540, we performed a cDNA analysis obtaining the same sequence that was deposited in the GenBank database and predicted as a hypothetical protein of 644 amino acids (acc. no. AY735746). Interestingly, the genes of AtTOP3α and AtRMI1 (At5g63540) are located in close vicinity on chromosome V, spaced by only 130 kilobases. We characterized two independent mutant lines of At*RMI1*. The first line, *rmi1-1* (SALK_93589), shows a deletion of approximately 2 kb starting in the first exon. It therefore virtually resembles a true knockout because more than 60% of the entire gene is lost (Figure 1B). Only a truncated version of the RMI1 mRNA is produced in the *rmi1-1* line (Figure S1B). The second line, *rmi1-2* (SALK_094387), harbours a duplicated T-DNA that has two left borders pointing outwards in the 5th exon and is accompanied by a small deletion of 12 nt (Figure 1B). We did not detect any expression in the *rmi1-2* line spanning the insertion site (Figure S1B).

Both lines show a similar phenotype, but not to the same extent. Both *rmi1-1* and *top3α-2* are completely sterile and severely impaired in male and female meiotic divisions, while *rmi1-2* shows a reduced fertility, producing about half the number of seeds as wild type plants. By analyzing meiotic anaphases, we found a defect in 51 out of 230 figures analyzed (20.8%). In the wild type, however, we detected only about 6% abnormalities (11/193). Thus, it seems that only *rmi1-1* can be regarded as a null mutant, at least with respect to meiosis.

### Defining the Role of AtRMI1 in Somatic Cells

To characterize the role of AtRMI1 in somatic cells, we performed a sensitivity assay towards genotoxic agents and changes in HR to detect defects in mitosis. Both *rmi1-1* and *2* failed to exhibit mitotic defects (Table 1). Our tests with the *rmi1-2* mutant revealed that the plants are more sensitive to DNA damaging agents MMS and cisplatin, to a similar extent as the *recq4A-4* mutant (Figure 3A and B). No sensitivity to camptothecin was detectable (Figure 3C). Moreover, tests performed with the *rmi1-1* mutant in the IC9C background revealed the same kind of HR behaviour observed in the *top3α* and the *recq4A* mutants. The loss of RMI1 results in hyper-recombination under standard growth conditions, however, no significant difference was observed after the induction of DSBs with bleomycin in wild-types (Figure 4). Thus, similar to TOP3α and RECQ4A, RMI1 is involved in the suppression of spontaneous recombination but not in interchromosomal homologous DSB repair. It therefore seems that in plants, a fully functional RTR complex is present and required for repair of certain kinds of DNA damage and crossover-suppression in somatic cells.

### The Role of AtRMI1 in Meiosis

**Figure 6:**
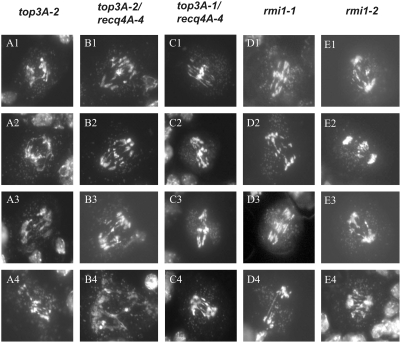
Examples of anaphase I in the mutant lines visualized by DAPI staining of developing pollen. Several anaphase I chromosome preparations of *top3α-2* and *rmi1-1* mutant lines are shown in comparison to the double mutants of *top3α-1* or *2* and *recq4A-4*.

Cytological analyses of AtRMI1 revealed similar kinds of meiotic defects in both lines. As for *top3α-2*, changes in meiotic progression were first detectable in the late prophase, appearing as fragmented DNA and more than five bivalent structures (Figure 5 E2). The most impressive meiotic defect is visible at anaphase I. In *top3α-2* mutants, most of the chromosome mass reached the pole in fragmented form, however, the greatest amount of DNA in *rmi1-1* remained at the position of the metaphase plate (Figure 5 E4). In both mutants as well as in the double *top3α* and *recq4A* mutants, an increased amount of entangled chromosomes that contained unresolved connections and were torn apart during anaphase I were observed. Furthermore, severe chromosome fragmentation occurred and long bridges between the chromosomes were visible (Figure 5 B3 and D4). Representative examples of anaphase I structures observed in the *top3α-2*, both *top3α* mutants in *recq4A-4* backgrounds as well as in *rmi1-1* and *2* are illustrated in Figure 6. Taking the number of visible fragments into account, it was clear that most chromosomes were broken more than once. The decondensation of chromosomes took place during telophase I, and like the *top3α-2* mutant, the *rmi1-1* mutant did not progress any further. The same effect was observed in ∼20% of the meiotic stages that were impaired in the hypomorphic *rmi1-2* line. Our results clearly demonstrate that both TOP3α and RMI1 are essential for the proper resolution of recombination intermediates during the first meiotic division.

## Discussion

### Different Functions of the RTR Complex

Very little is known about the biological function of TOP3α in higher eukaryotes. This is because that deletion or disruption of TOP3α in mammals, worms, and insects leads to severe phenotypes comparable to the Arabidopsis T-DNA insertion line *top3α-1* [8]–[10],[12]. We were able to restore the viability, but not the fertility, of this mutant using a *recq4A* background, suggesting that TOP3α might also be essential for meiosis. The characterization of a second *TOP3α* mutant with a less severe somatic phenotype has enabled us to define specific functions of the protein during mitosis and meiosis. Our finding that the phenotype could be complemented by the wild-type gene to the same extent as for the *top3α-1* mutant suggests that *top3α-2* can be regarded as hypomorphic.

**Figure 7:**
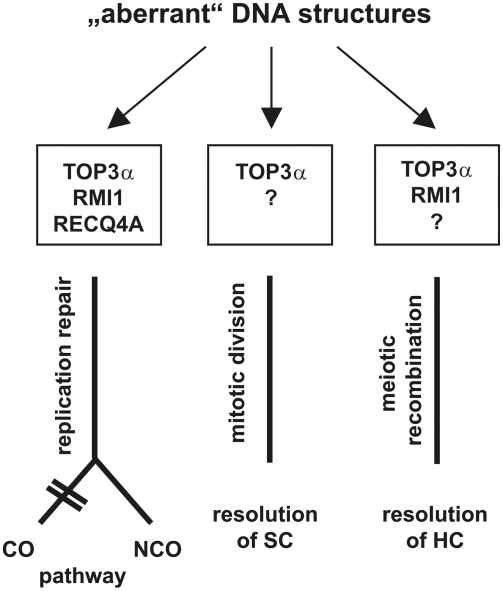
Three different pathways to remove aberrant DNA structures in which AtTOP3α is involved. In replication repair, the RTR complex was involved in the suppression of CO recombination by removing recombinogenic DNA structures. In mitotic division, only TOP3α was necessary for proper segregation of the sister chromatids (SC). Neither RECQ4A nor RMI1 were required for this segregation. During meiotic recombination, both TOP3α and RMI1 were essential for the resolution of homologous chromosomes (HC).

Our experiments clearly demonstrate that AtTOP3α is involved in at least three different pathways: i) replication-dependent DNA repair and suppression of CO-recombination in somatic cells, ii) proper resolution of replication intermediates during mitosis, and iii) resolution of homologous chromosomes during meiosis. Moreover, the results from single and several double mutants enable us to define the processes in which TOP3α is acting in concert with its partners of the predicted plant RTR complex, namely RECQ4A and RMI1 (Figure 7).

Our results also show that a functional RTR complex is present in plants as previously demonstrated for other eukaryotes. First, homologues of all three genes are not only present in the genome of Arabidopsis but also distantly related plants, such as poplar, rice and moss (according to our database searches). We demonstrated that the three single mutants of the respective genes in Arabidopsis exhibited a similar sensitivity to genotoxic agents that methylate or crosslink DNA. This clearly shows that the RTR-like complex is involved in somatic DNA-damage repair in *A. thaliana*. Moreover, this complex directs the repair of aberrant DNA-structures during replication into a pathway by which HR is suppressed [12]. The formation of DNA structures that could enhance HR is therefore either prevented, interrupted or even dissolved during later steps as it has been shown for the RTR complex in yeast and animals [13],[15],[18],[30]. This CO-suppression function conserved in plants is demonstrated by the fact that insertion lines of all three RTR members exhibit an enhanced level of HR without induction of DSBs, indicating their involvement in a replication-associated repair pathway.

Second, mutation of RECQ4A rescues the lethal phenotype of *top3α-1*. This phenomenon is similar to that in *S. cerevisiae*. Here, mutation of the Slow Growth Suppressor 1 (Sgs1) rescues the very slow growth of a *top3* mutant [3]. The double mutant *recq4A-4/top3α-1* or *2*, however, exhibits the same defects in mitotic division as the *top3α-2* single mutant, indicating that a functional TOP3α is always required for mitosis. Our analyses revealed no mitotic defects for the *recq4A-4* or *rmi1-1* and *rmi1-2* mutants, indicating that the RTR complex is not necessary for this function of TOP3α. Thus, TOP3α possesses a unique role during mitosis, which was sustained by the observation that *top3α-2* was the only line that showed enhanced sensitivity in the camptothecin treatment assay. This chemical specifically blocks replication forks by trapping TOP1 covalently bound to DNA [31],[32], which may indicate that AtTOP3α can at least partially substitute for a class IB topoisomerase.

Third, we showed that both TOP3α and RMI1 have an essential role during meiosis. Here, RECQ4A is either not involved or might be substituted by another helicase. This is supported by the findings that *recq4A-4* does not show any visible meiotic defects [12],[25]. Notably, besides the *recq4A* mutant, *recq4B* mutants and the double mutant *recq4A/recq4B* are also fully fertile, despite the fact that both genes are most closely related to the mammalian BLM homologue [12]. The data presented in our manuscript, however, do not exclude that RECQ4A is involved in the resolution of recombination intermediates during meiosis as it has been recently shown for the yeast homolog Sgs1 [33],[34]. It seems that during meiosis, both TOP3α and RMI1 are essential for the proper resolution of homologous chromosomes, as mutations in both genes show very similar meiotic defects. The most intriguing feature is that TOP3α as well as RMI1 mutants do not progress through meiosis past telophase I. This is in contrast to all meiotic mutants described in plants to date, which all complete meiosis even if chromosome fragmentation occurs [35]–[40]. The telophase I arrest found here is most likely due to the fact that the homologous chromosomes (HC) are not separated properly, and a signal to proceed with meiosis, which may require resolution of the chiasmata between HC, is not given. An alternative explanation might be that the meiotic abrogation occurs due to the extensive damage that may mechanically hinder the progression of meiosis. Conversely, severe damage also accumulates in other meiotic mutants, which are still able to enter meiosis II.

### Role of TOP3α/RMI1 during Meiotic Recombination

Our data unambiguously demonstrate that both TOP3α and RMI1 are required in the late steps of meiotic recombination. It is difficult, however, to define their particular role in detail. The lack of either TOP3α or RMI1 clearly leads to plain sterility due to meiotic arrest during telophase I, as the proteins appear to be involved in the resolution of recombination intermediates. A major question of debate has been what the nature of Holliday Junction resolvases responsible to resolve the CO-structures during meiosis. It has been shown that at least two different complexes are present in most eukaryotes. One protein complex consists of the MUS81/EME1 endonuclease, and the other is associated with RAD51C and XRCC3 [41]–[43]. As it is generally assumed that such an enzyme complex should be able to resolve dHJs into COs, we are reluctant to assume that TOP3α/RMI1 are participating in CO-formation because both proteins are involved in the suppression of COs in somatic cells. Furthermore, topoisomerases of class I are not classical HJ resolvases. Together with the help of a helicase, dHJs can be transformed into hemicatenanes, which then can be resolved into two unlinked double strands by topoisomerase-mediated strand passage [44]. The classical DSB repair (DSBR) model predicts a dHJ as the key intermediate, whose resolution leads to either CO or nCO [45]. This theoretical prediction has been sustained later on the molecular level in yeast by 2D gel analysis, demonstrating that the dHJ is a major heteroduplex intermediate in yeast meiosis [44],[46]. A revised model of meiotic recombination was suggested in 2001. It was postulated that a major number, if not all nCO recombinants, do not arise from dHJs but rather from synthesis-dependent strand annealing (SDSA) using an intermediate D-loop [47]–[49]. The revised DSBR model involves an intermediate called single end invasion (SEI), which occurs at the transition state between D-loop and dHJ during yeast meiosis [48]. It was proposed that the CO-decision is made very early during leptotene/zygotene, probably by a differential loading of RAD51 or DMC1 [48],[50]. Taking the revised model into account, it is tempting to speculate that TOP3α/RMI1 may function at the resolution step of dHJs (Figure 7). Our hypothesis is that TOP3α/RMI1 acts as a type of safeguard system to resolve dHJs to nCO that were not resolved by a bona fide resolvase, even though they had been channelled into the CO pathway. This mode of dHJ resolution by a topoisomerase type I has was proposed long ago [51]. Schwacha and Kleckner also discussed this potential resolution of mature dHJs via a topoisomerase function as either an alternative to the modified DSBR model or as a backup mechanism for the resolution of an HJ that aberrantly persists beyond pachytene [44]. We favour the hypothesis that the resolution step in the CO pathway of meiotic recombination is either surprisingly error prone or rate limiting. Whenever HJ resolution fails, only the alternative dissolution of an HJ into nCO guarantees the progression of meiosis [47],[48]. Failure of the TOP3α/RMI1 function could lead to the persistence of HJs that would have been processed into hemicatenane structures. These structures, however, could only be cleaved by a topoisomerase and no longer by a resolvase. Thus, due to mechanical shearing of the irresolvable interlinked homologues, we observed the fragmentation of chromosomes and telophase I arrest. An alternative explanation would be that TOP3α is specifically required in meiotic chromosome condensation during or after recombination to remove torsional stress. If the enzyme is not functional, chromosome breakage may occur. We, however, disfavour this hypothesis as at least yeast TOP3, in contrast to TOP1 and 2, is insufficient to remove supercoils *in vitro* [52]. Moreover, it is not clear why an RMI1 homologue would be involved in such a function as, in contrast to TOP3α, it is not required for mitosis in Arabidopsis.

### Safeguarding Meiosis

Our results demonstrate that both genes, TOP3α and RMI1, are essential for proper meiotic progression, and if disturbed, result in fragmented chromosomes and meiotic arrest at telophase I. In an independent study, the group of Mathilde Grelon (Chelysheva et al., submitted to PLoS) identified RMI1-deficient plants in a screen for sterile Arabidopsis mutants. Their detailed analyses of the meiotic role of RMI1 are in complete accordance with our results presented here. Moreover, they showed that RMI1 is not required for synaptonemal complex formation. Analyzing crosses between *rmi1* and *dmc1* or *rad51*, they were further able to demonstrate that the protein is required for meiotic DSBR using either the homologue or the sister chromatid as a template.

Thus, two independent studies strongly indicate that higher eukaryotes possess a safeguard mechanism to guarantee the untangling of homologous chromosomes in the late meiotic prophase. This process seems to be conserved in yeast as it has been demonstrated that the mutation of the unique TOP3 of *S. cerevisiae* leads to both mitotic defects and sterility [3],[53]. Although no detailed cytological analysis was performed, it could be demonstrated that the phenotype was the result of a defect in meiotic recombination.

It is tempting to speculate that the role of TOP3α/RMI1-1 in meiosis is functionally related to their roles in suppression of sister chromatid exchanges (SCE) during replication in mitosis. Nevertheless, both processes seem to be different because in plants, RECQ4A is also involved in SCE suppression along with TOP3α and RMI1. The fact that *recq4a* mutants are fertile does not necessarily imply that RECQ4A has no role in meiosis. It has been recently shown that SGS1, the unique RecQ homologue of yeast, is indeed directly involved in meiosis by suppressing formation of joint molecules comprising three and four interconnected duplexes [54]. Important aims of future research will therefore be to elucidate the role of AtRECQ4A in meiois as well as to identify the putative helicase that, according to our hypothesis, might be involved in the dissolution of dHJs in concert with AtTOP3α and AtRMI1 in meiosis.

## Materials and Methods

### Molecular Characterization of the T-DNA Insertion Lines and RACE

The genotyping of the different mutants was perfomed as described [12] using primers flanking the T-DNA insertions (Figure 1A and C and Table S1). Interestingly, the 2 kb deletion found in *rmi1-1* seems to have occurred during propagation of the seeds at the SALK institute. Indeed our seed sample of SALK_093589 contained a mixture of two different genotypes. Besides the allele with the deletion, we found also a T-DNA inserted as described by SALK. Since we were afraid that the T-DNA insertion might be unstable during propagation we decided to perform our experiments with the deletion mutant. This line was stable in its phenotype for at least 4 generations in our hands. The T-DNA integration sites and the deletion in *rmi1-1* were determined via PCR using primer combinations specific for the left border of the respective T-DNA and genomic sequences within the respective gene (Figure 1A; primer 2, 2R, and LB; Figure 1C; primer 4, 5R and LB1).

The Rapid Amplification of cDNA Ends (RACE) was carried out according to Matz et al. (1999) [22].

### Mutagen and HR Assays

Mutagen and HR assays using the IC9C reporter line and the respective T-DNA insertion lines were perfomed as described [12]. Specific adaptions concerning these two general assays for sterile plants were as follows below

### Mutagen Assay, Modifications for Sterile Plants

Seeds were sterilized using 4% NaOCl solution. After two weeks of growth on solid germination medium (GM), plantlets were transferred into six-well-plates containing 5 ml of liquid GM medium for each untreated control, or 5 ml GM including the mutagen samples, respectively. Five plants were used for each well. For the *top3A*-2 mutant only homozygous plants were picked with regard to their visible growth phenotype. The *top3A-2* phenotype was confirmed via PCR screening. After 14 more days in the growth chamber, the fresh weight of each sample was determined using a fine scale balance. To avoid any aberrations resulting from excessive liquid, the plant material was dabbed off with paper towels before weighting. The mutagen assays were carried out at least four times for each mutagen.

### HR Assay, Modifications for Sterile Plants

The assay was carried out under sterile conditions, transferring in each case 20 2-week old plantlets from solid into liquid GM medium (10 ml). Bleomycin was added dissolved in GM the next day in a final concentration of 5 µg/ml. After 6 days in the growth chamber the seedlings were transferred into staining solution. For the *rmi1-1* and *top3A-2* lines, homozygous plants were identified by means of PCR screening and only the data obtained from these seedlings were included in the analysis.

### Analysis of Germ Cells and Complementation

Meiotic and mitotic figures were analysed as described [28].

To rescue the different phenotypes observed in the *top3α-1* and *2* mutant lines, we generated a complementation construct of 8711 bp representing the entire gene of TOP3α. It included 1174 bp of the promoter region, 114 bp 5′-UTR, 6699 bp between start and stop codon, 203 bp of the 3′-UTR and 521 bp of the terminator region. The genomic region was amplified in two parts using linker-primers and a proofreading polymerase (Phusion-*Taq*, New England Biolabs). The fragments cloned into the vector pPZP221 [55] were sequenced, subsequently combined via usage of an internal *Nde*I restriction site and transformed into the heterozygous *top3α-1* and *2* plant lines. Successful transformed plants showing gentamycin resistance have been screened afterwards for their respective genotype concerning the TOPα gene. For this screening we used primers that were specific for the genomic locus only (Table S2).

## Supporting Information

Figure S1 mRNA expression of the respective genes interrupted by T-DNA insertions.
(0.38 MB DOC)

Figure S2 DAPI staining of different meiotic stages during pollen development in the mutant line At*recq4A-4*.
(1.79 MB DOC)

Table S1 Different primer used for RT-PCR and screening analyses of the T-DNA insertion lines.
(0.03 MB DOC)

Table S2 Different primer and linker-primer used for the complementation analyses.
(0.02 MB DOC)
