# Supplementary material for: Topoisomerase 3α and RMI1 Suppress Somatic Crossovers and Are Essential for Resolution of Meiotic Recombination Intermediates in Arabidopsis thaliana
Source: PLoS Genet. 2008 Dec 19;4(12):e1000285. doi: 10.1371/journal.pgen.1000285 (PMC2588661; doi:10.1371/journal.pgen.1000285)
Supplement: Figure S1 — mRNA expression of the respective genes interrupted by T-DNA insertions. (0.38 MB DOC) [file pgen.1000285.s001.doc]

**M bf sp bh bf sp bh bf sp bh M**

**Col-0**

***top3α-1***

***top3α-2***

**A**


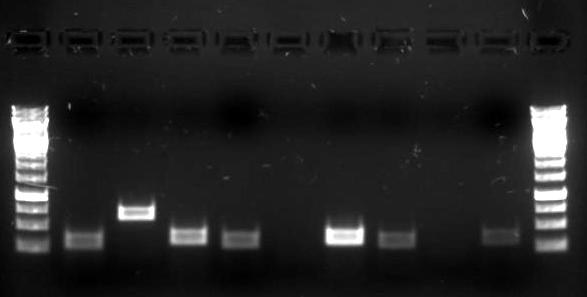


**before T-DNA**

**spanning T-DNA**

**behind T-DNA**

**M 1 2 3 4 5**

**1 2 3 4 5**

**1 2 3 4 5 M**

**B**


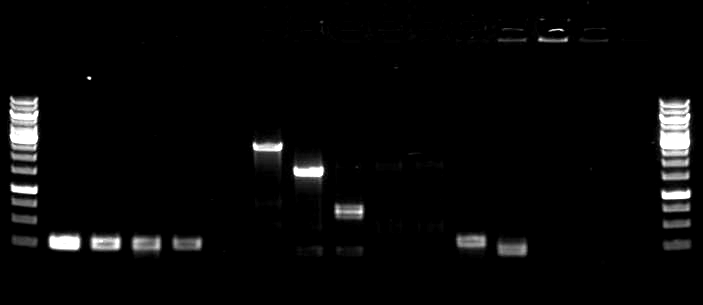


**Figure S1**  mRNA expression of the respective genes interrupted by T-DNA insertions. The mRNA expression of the respective T-DNA knockouts lines has been tested using primers located either before (bf), spanning (sp) or behind (bh) the T-DNA insertion locus. (**A** and **B)** M= DNA size marker (1 kb ladder, Fermentas). (**B**) 1. Col-0 genomic DNA control; 2. Col-0 cDNA; 3. *rmi1-1* cDNA; 4. *rmi1-2* cDNA; 5. H2O control. The locations of the primers used for PCR are given schematically in main Fig. 1, primer sequences are shown in Supplemental Table 1. None of the mutants exhibited expression spanning over the insertion locus except *rmi1-1* which shows a truncated cDNA (**B**, spanning: line 3). In front of the insertion sites comparable amounts of cDNA, as in the Col-0 control, were detected for all mutant lines and only in case of both *top3α* lines also normal or slightly elevated expression could be detected (**A**, lanes bh) whereas both *rmi1* lines did not show expression behind the respective insertion site and (**B**, lanes behind 3 and 4).
