# Supplementary material for: Topoisomerase 3α and RMI1 Suppress Somatic Crossovers and Are Essential for Resolution of Meiotic Recombination Intermediates in Arabidopsis thaliana
Source: PLoS Genet. 2008 Dec 19;4(12):e1000285. doi: 10.1371/journal.pgen.1000285 (PMC2588661; doi:10.1371/journal.pgen.1000285)
Supplement: Figure S2 — DAPI staining of different meiotic stages during pollen development in the mutant line Atrecq4A-4. (1.79 MB DOC) [file pgen.1000285.s002.doc]

***recq4A-4***

### Col-0


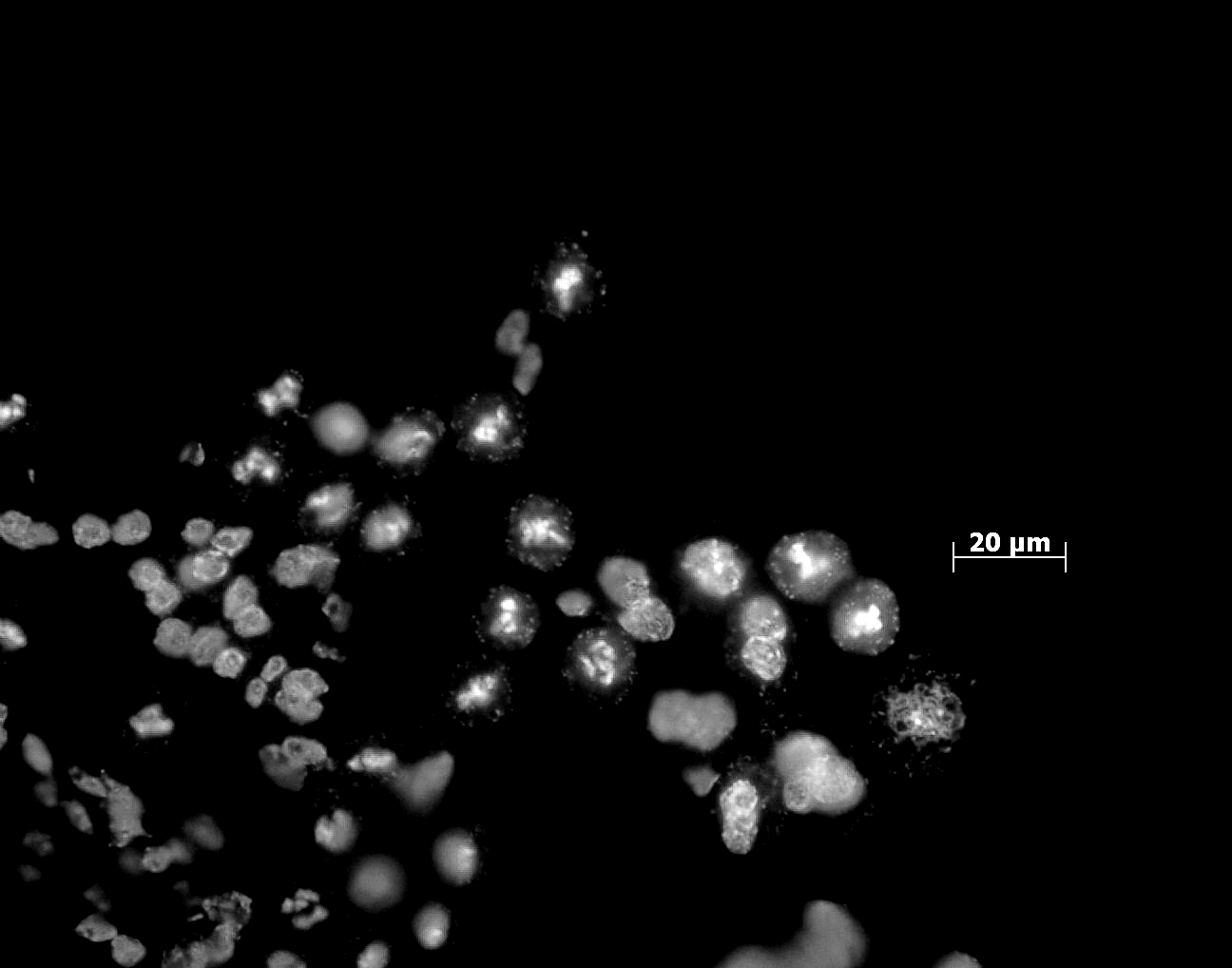

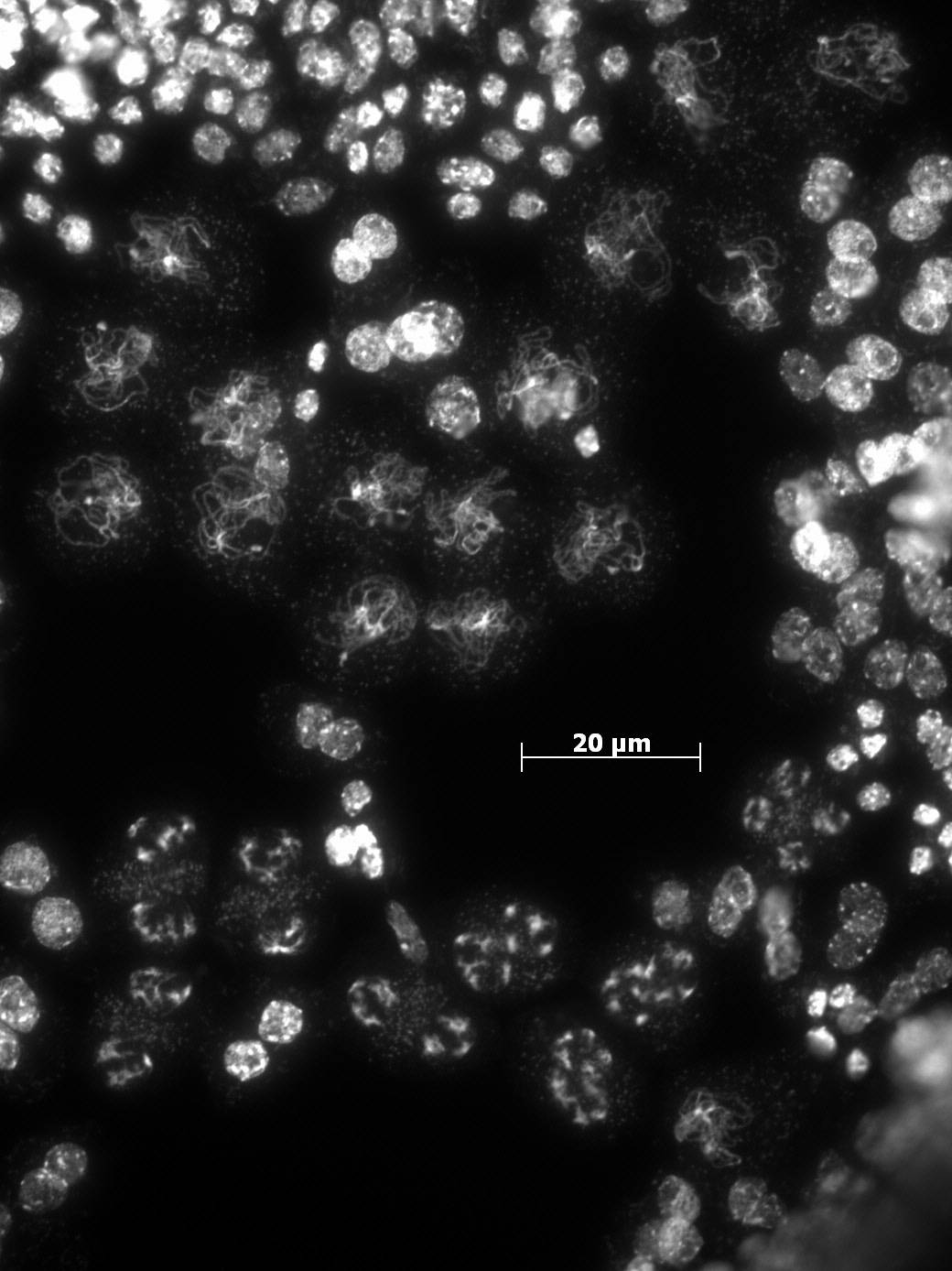


**pachytene**

**A1**

**B1**


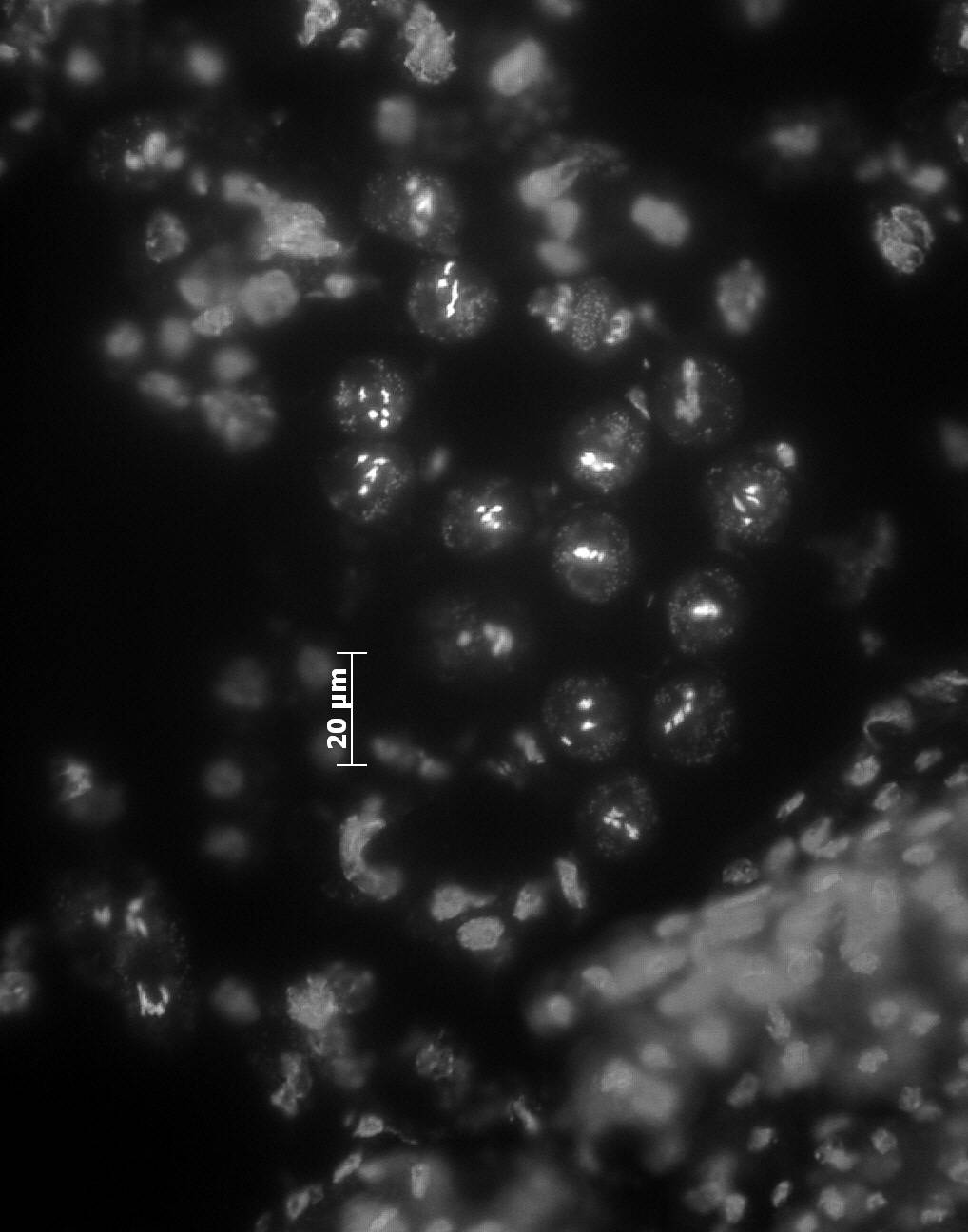

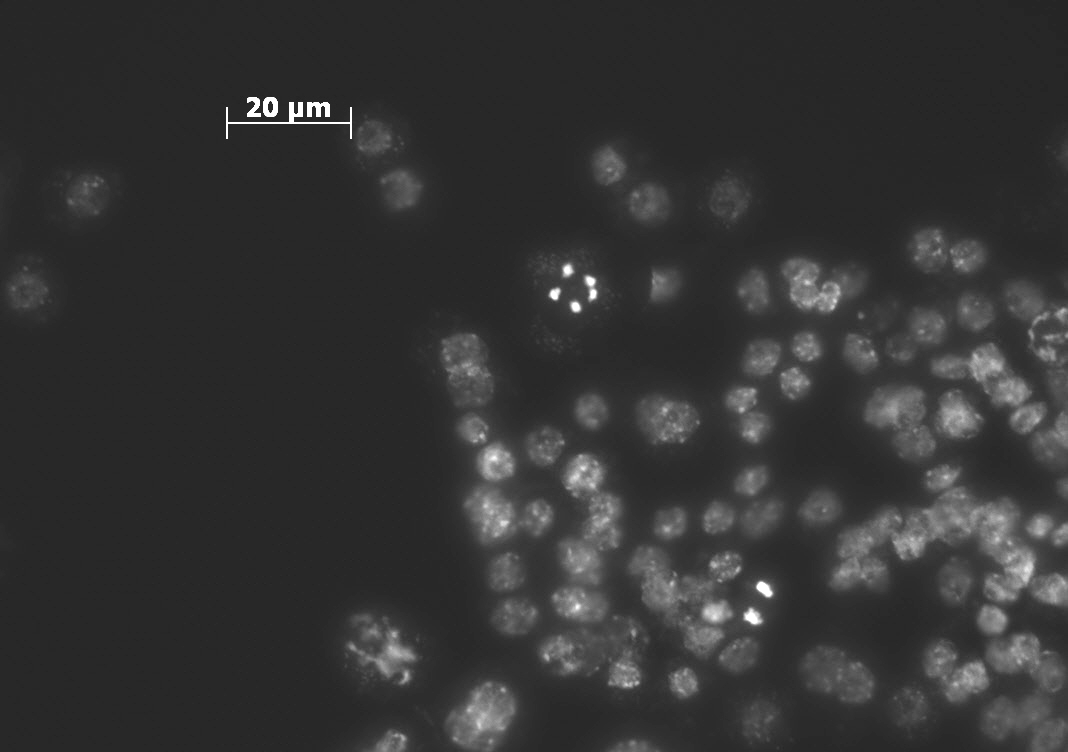


**diakinesis**

**A2**

**B2**

**A3**

**B3**


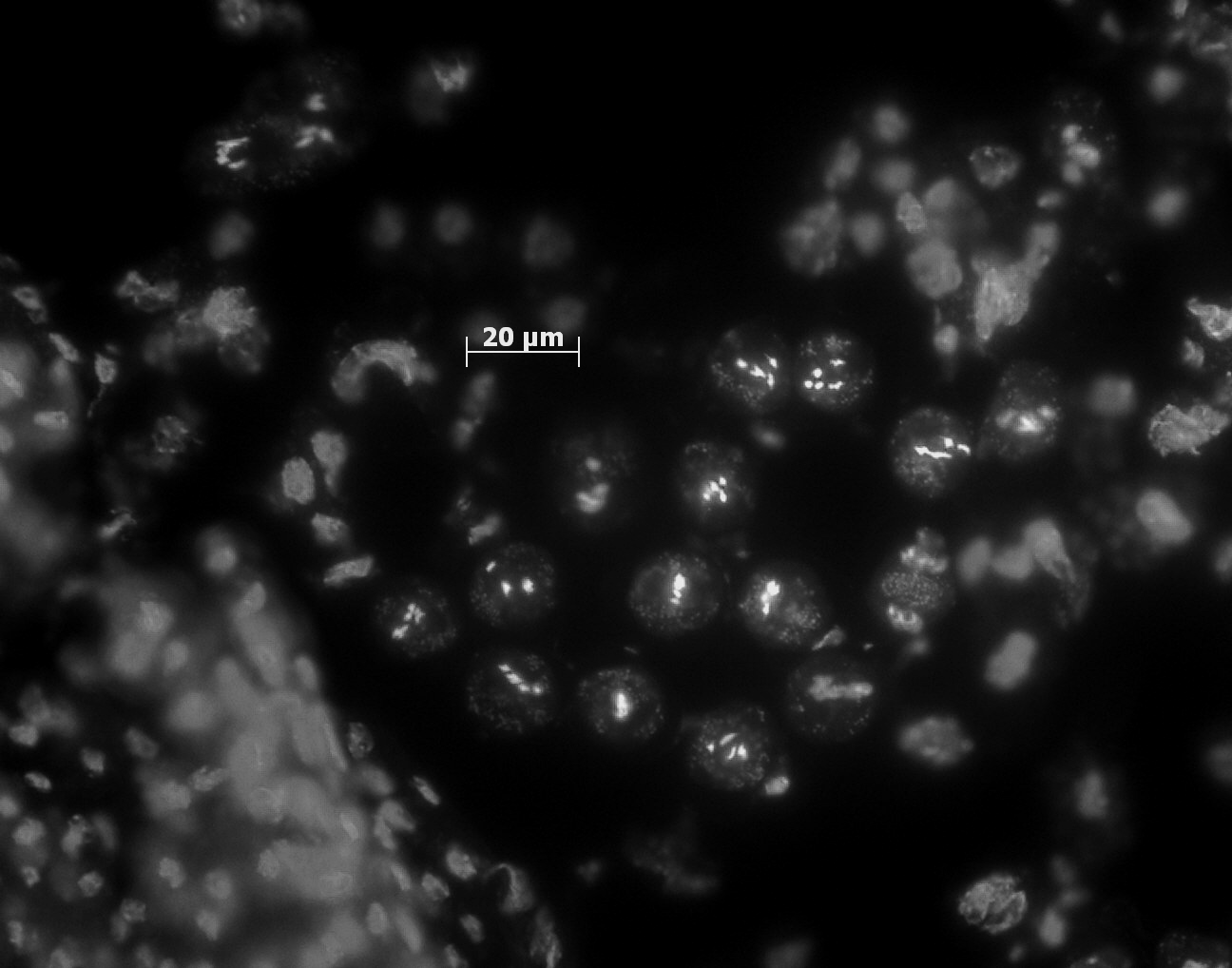

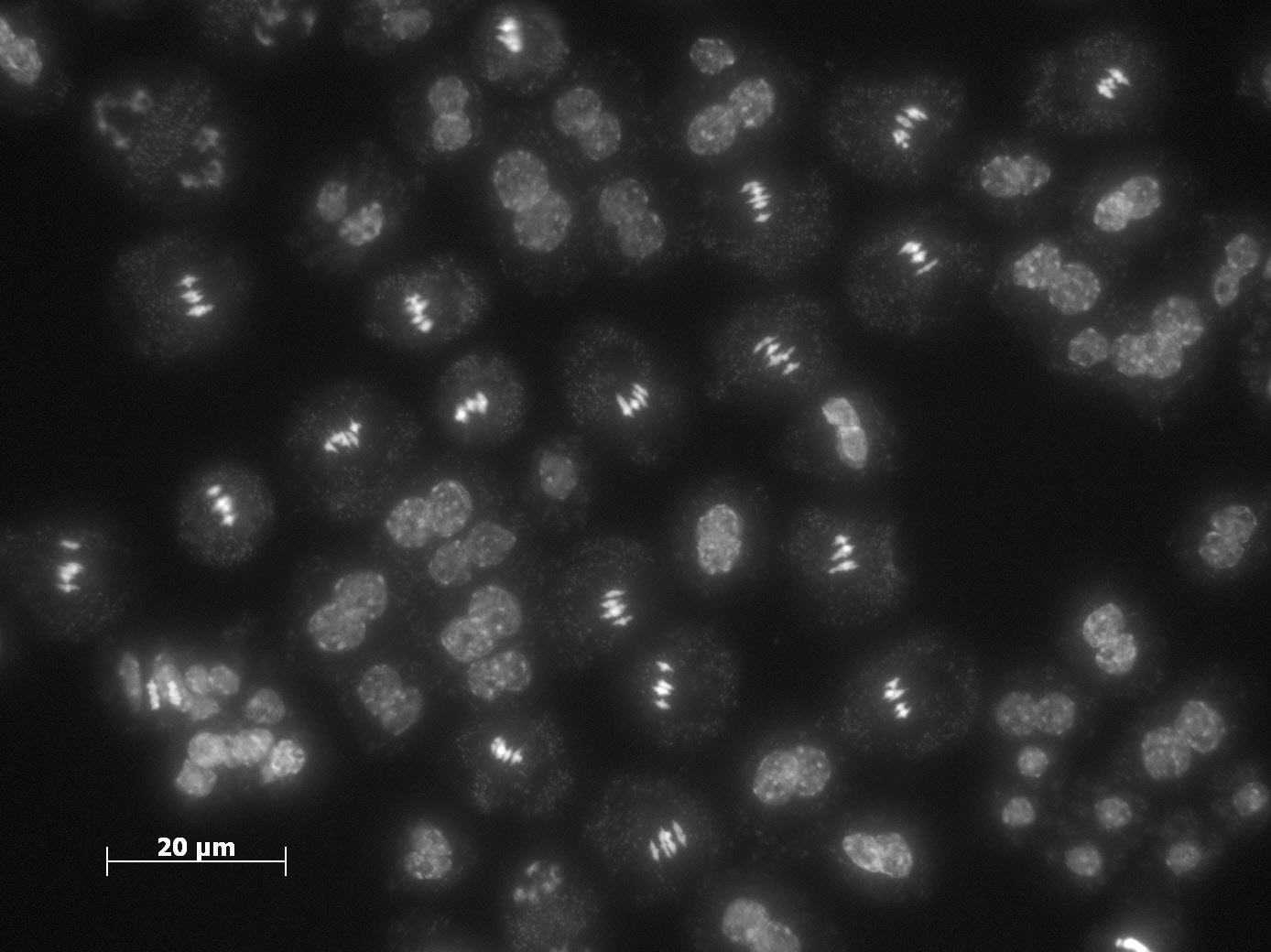


**metaphase 1**


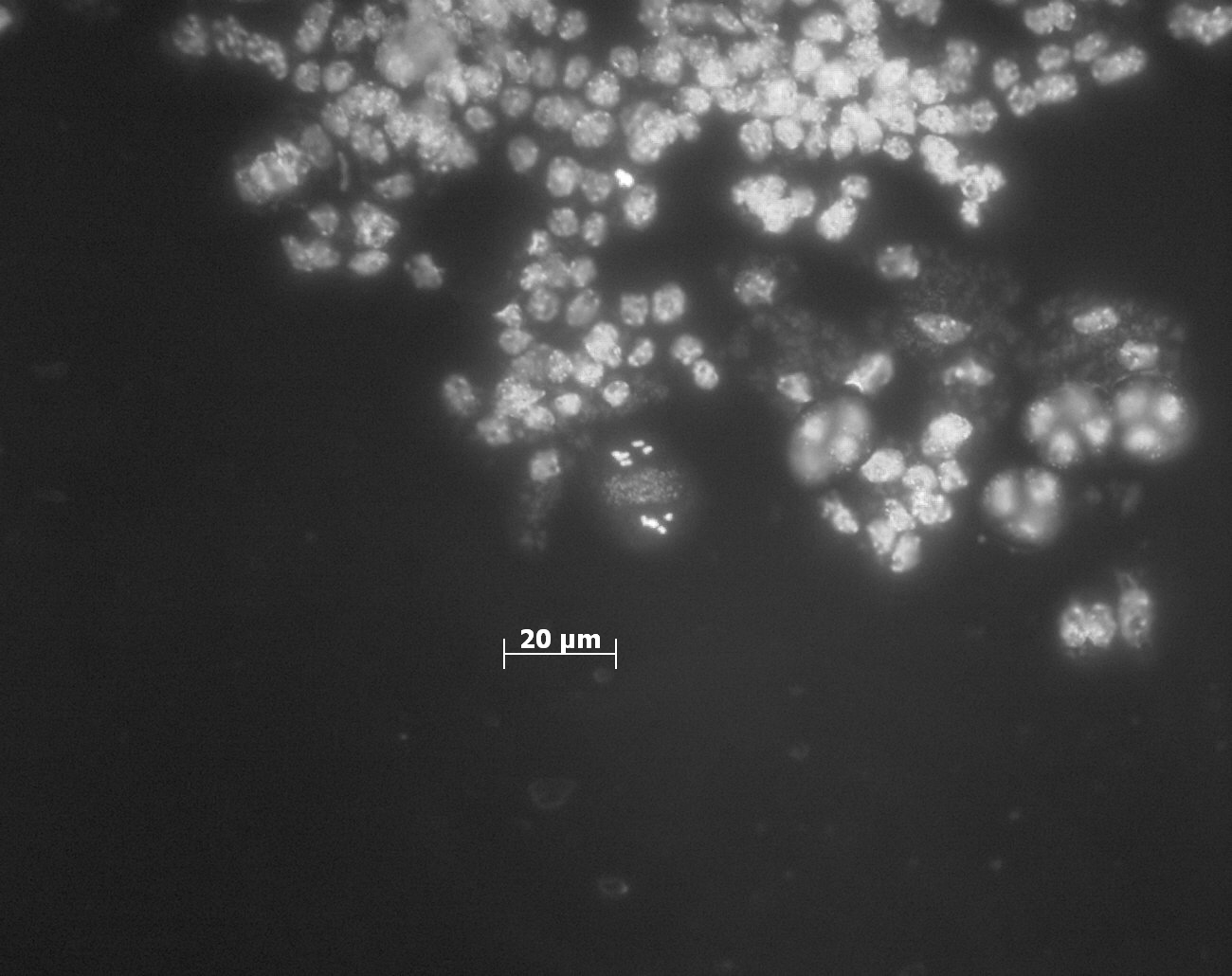

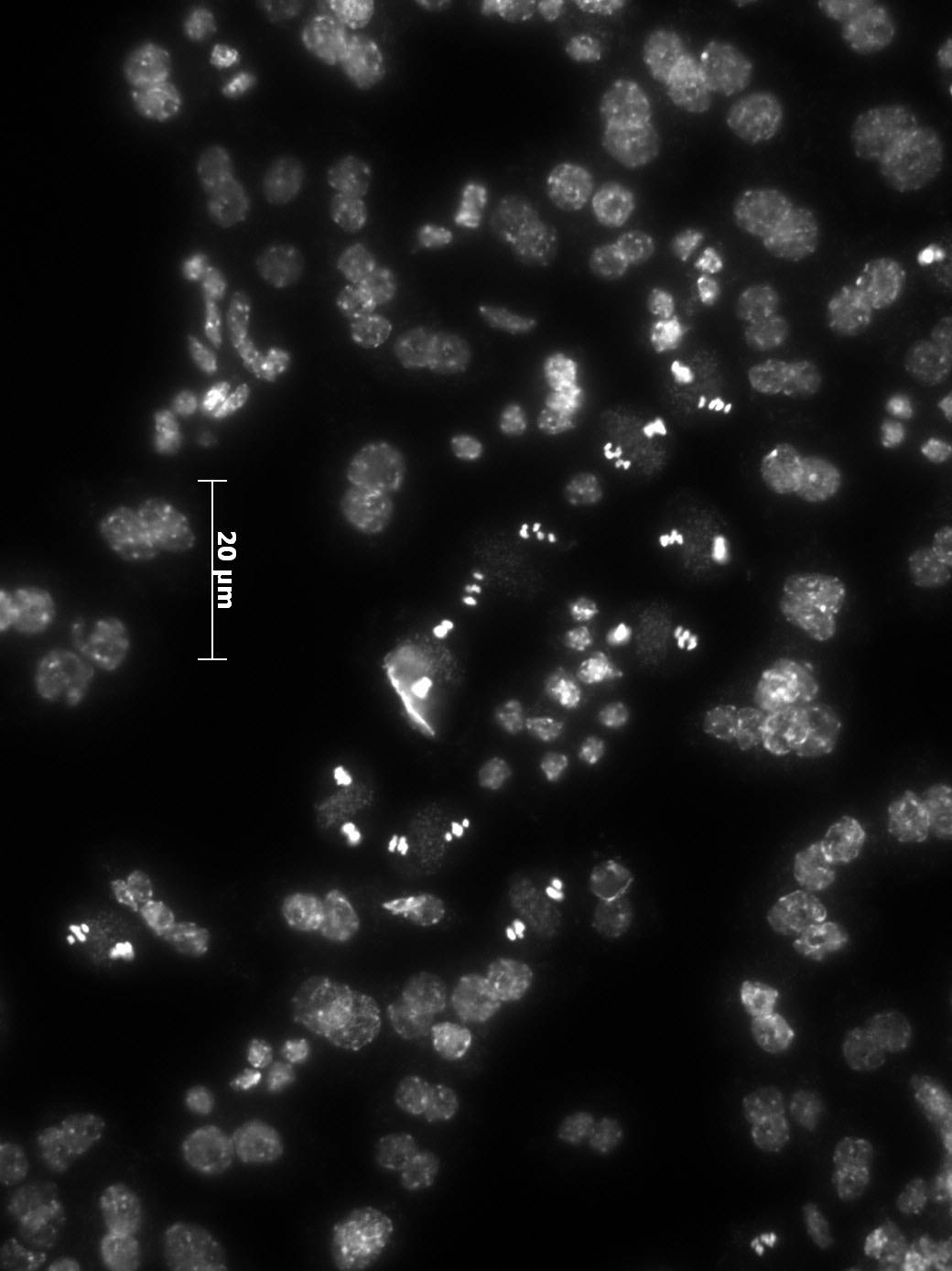


**anaphase I**

**A4**

**B4**


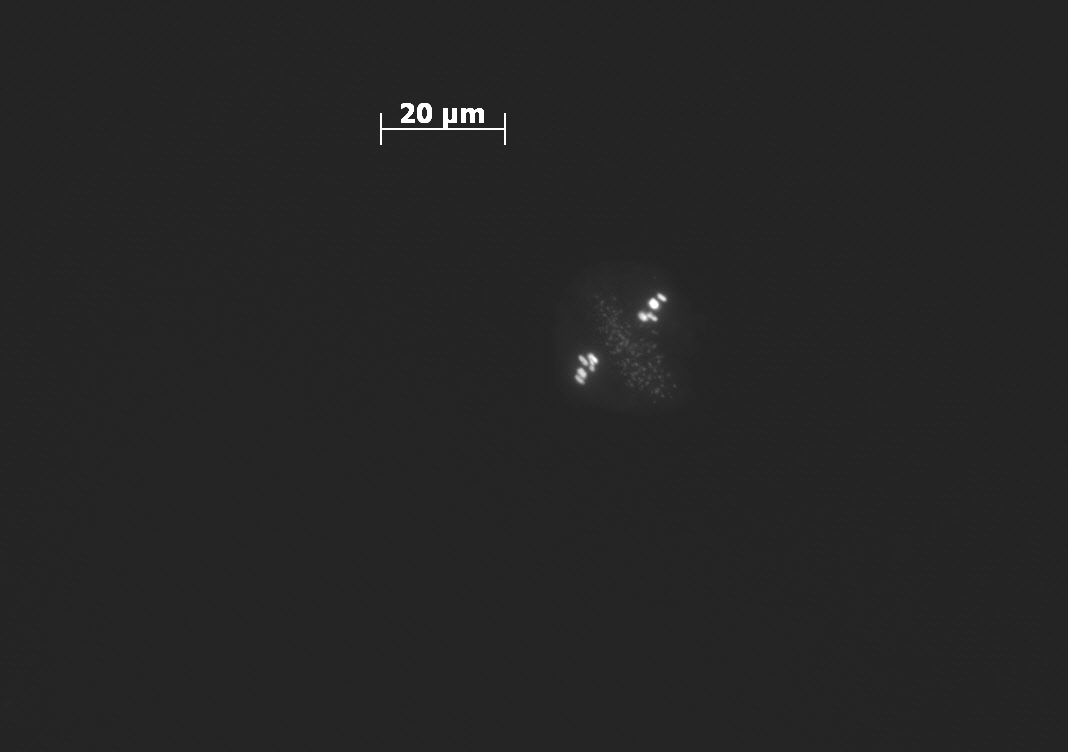

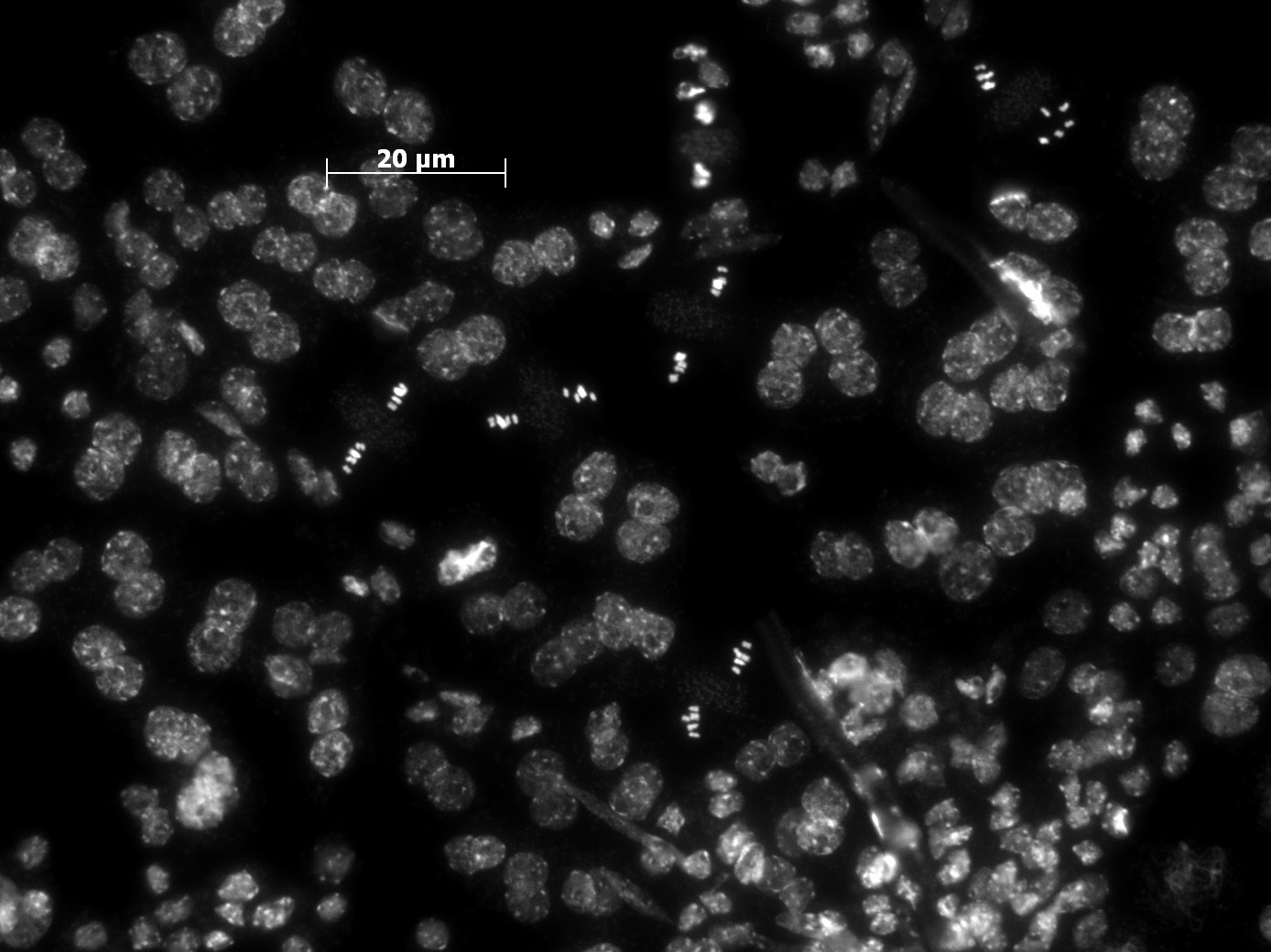


**telophase I**

**A5**

**B5**


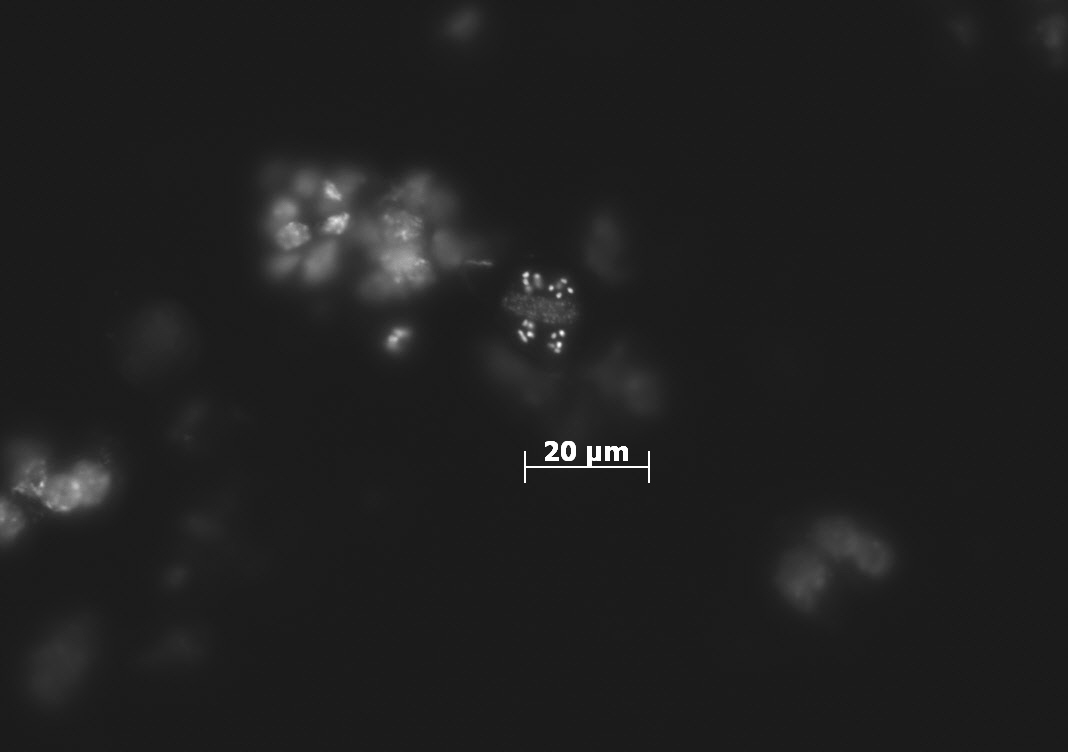

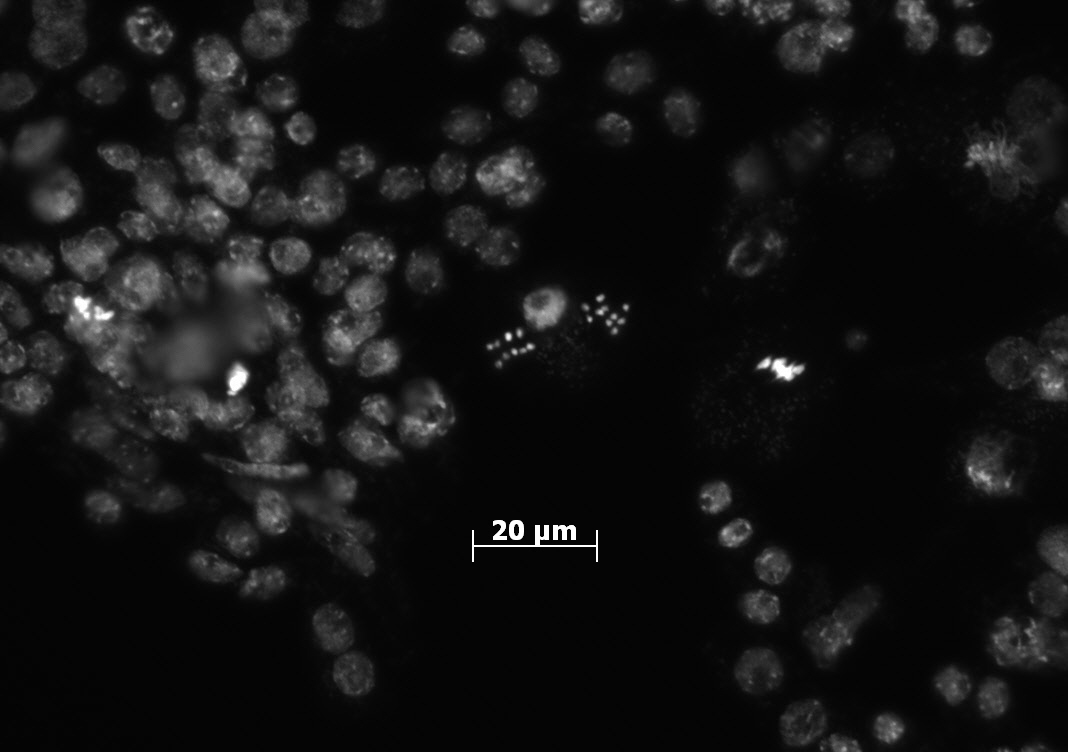


**anaphase II**

**A6**

**B6**


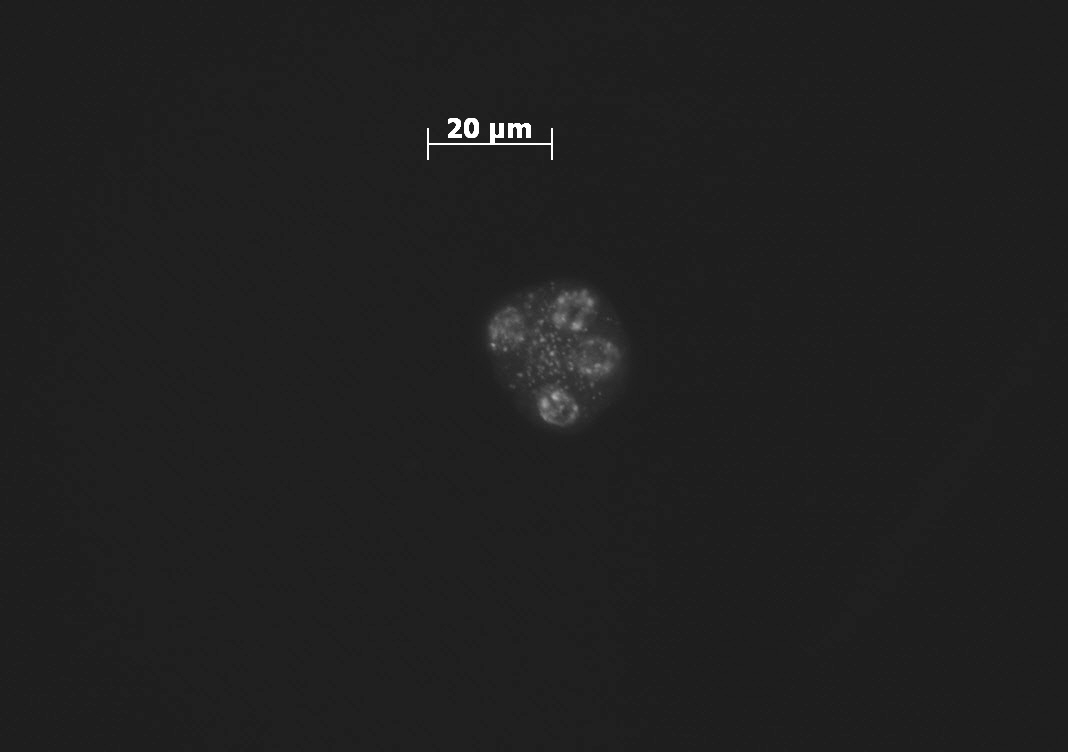

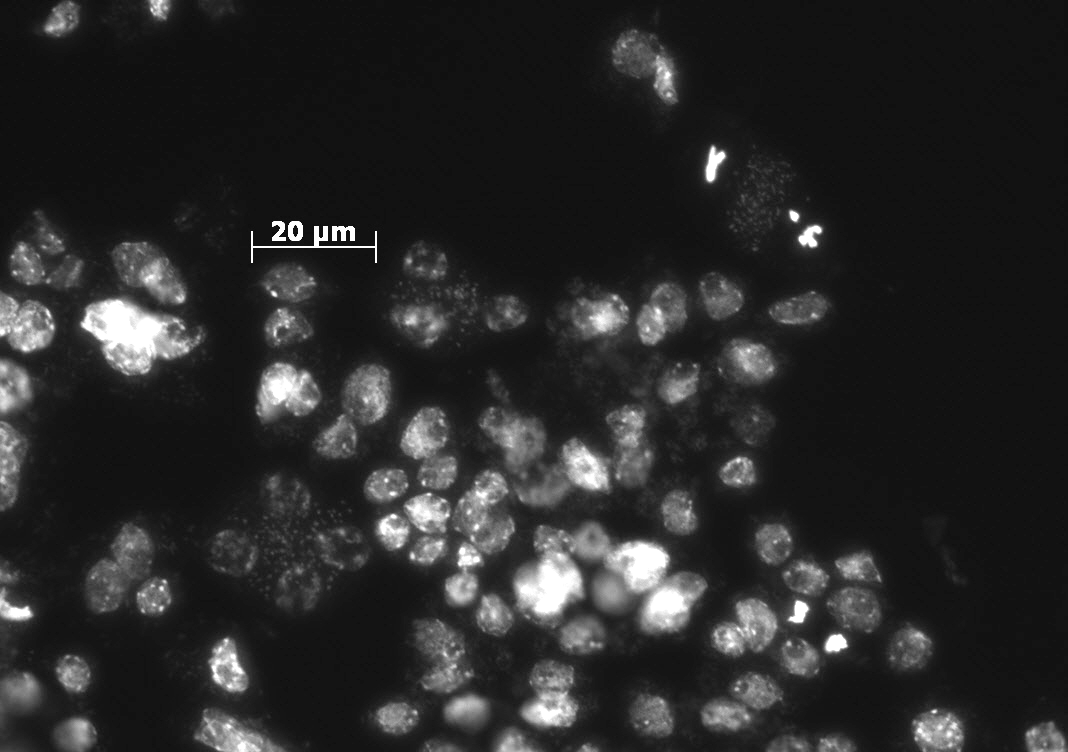


**telophase II**

**A7**

**B7**

**Figure S2** DAPI staining of different meiotic stages during pollen development in the mutant line At*recq4A-4*. Wild type meiosis (A1-7) is characterized by synapsis of homologous chromosomes and the formation of bivalents during pachytene stage of prophase (A1). During diakinesis (A2) the chromosome pairs condense for the first meiotic division. At metaphase I homologues congress at the metaphase plate (A3). During anaphase I the homologous chromosomes separate to the poles (A4) and decondense in telophase I (A5). During anaphase II the chromatids slightly condense and separate (A6) followed by another decondensation phase in telophase II and end up in tetrads (A7). The T-DNA insertion line *recq4A-4* (B1 to B7) shows no differences in comparison to meiosis during pollen development cells in the wild type Col-0.
