# Supplementary material for: Topoisomerase 3α and RMI1 Suppress Somatic Crossovers and Are Essential for Resolution of Meiotic Recombination Intermediates in Arabidopsis thaliana
Source: PLoS Genet. 2008 Dec 19;4(12):e1000285. doi: 10.1371/journal.pgen.1000285 (PMC2588661; doi:10.1371/journal.pgen.1000285)
Supplement: Table S1 — Different primer used for RT-PCR and screening analyses of the T-DNA insertion lines. (0.03 MB DOC) [file pgen.1000285.s003.doc]

**Table S1** Different primer used for RT-PCR and screening analyses of the T-DNA insertion lines.

| Primer | locus | sequence 5´- to 3´- | Tm [°C] |
| --- | --- | --- | --- |
|  | At*TOP3α* |  |  |
| 1 |  | GATATTAAGAAGACATTGGAGG | 60 |
| 1R |  | CAGCCTCAGCAAACAATTG | 56 |
| 2 |  | CATTACCTAGCATGTGTTTC | 57 |
| 2R |  | TTCCTGAGTGCCATATCTG | 56 |
| 3 |  | ACGAATTGTCCCTCACGGG | 60 |
| 3R |  | GCTCTCCAGTTGCAGAGAC | 60 |
|  | At*RMI1* |  |  |
| 4 |  | TGCGTAGACGGCGCCTG | 58 |
| 4R |  | TCCGACGGATAAACCGGA | 56 |
| 5R |  | GTTGTAATCTGTCAACCAAC | 56 |
| 6 |  | CTGGAGTGAAGAAATTCCAG | 58 |
| 6R |  | GTCAGCGAGAAACAACTG | 54 |
|  |  |  |  |
| LB | GABI T-DNA | GACCATCATACTCATTGCTG | 60 |
| LB1 | SALK T-DNA | TCGGAACCACCATCAAACAG | 58 |

The oligonucleotides used as primers for the RT-PCR possess a similar Tm (melting temperature) which has been calculated using the formula: [2*(AT) + 4*(GC)].
