# Supplementary material for: Topoisomerase 3α and RMI1 Suppress Somatic Crossovers and Are Essential for Resolution of Meiotic Recombination Intermediates in Arabidopsis thaliana
Source: PLoS Genet. 2008 Dec 19;4(12):e1000285. doi: 10.1371/journal.pgen.1000285 (PMC2588661; doi:10.1371/journal.pgen.1000285)
Supplement: Table S2 — Different primer and linker-primer used for the complementation analyses. (0.02 MB DOC) [file pgen.1000285.s004.doc]

**Table S2** Different primer and linker-primer used for the complementation analyses.

| Primer name | sequence 5´- to 3´- | specific part [nt] |
| --- | --- | --- |
|  |  |  |
| T3A-UTR-FW | GATTGTACAAGCCTGACCA | 19 |
| T3A-UTR-FW-SalI | acgcgtcgacGATTGTACAAGCCTGACCA | 19 |
| T3A-MID-R1 | TGGGAGAGCAATATTAGCC | 19 |
|  |  |  |
| T3A-3 | GGTGGAGGAACAAACAAGAC | 20 |
| T3A-UTR-REV | CAGGAGACGGCCTAATCA | 18 |
| T3A-UTR-REV-SacI | gctagagctcCAGGAGACGGCCTAATCA | 18 |
|  |  |  |
| T3A-C-SCR-FW | TGTGAGATTGTACAAGCCTG | 20 |
| T3A-C-SCR-Rev | CTACAACAACTCCTTCGATG | 20 |

The oligonucleotides used as linker primers for the complementation analyses contained restriction sites at their 5´-end. The specific part of the oligonucleotides is shown as capital letters; the linker region as small letters; the respective restriction site as underlined small letters. SCR = primers used for screening of the genotype.
